# Development of an interprofessional person-centred care concept for persons with care needs living in their own homes in Germany (*interprof* HOME): a mixed methods study

**DOI:** 10.1186/s12875-025-03098-0

**Published:** 2025-11-15

**Authors:** Britta Tetzlaff, Ana L. Mazur, Uta Sekanina, Tilman Huckle, Richard Dano, Carolin Höckelmann, Marilena Diel, Lea Bremer, Anja Kühn, Anna-Marie Romanski, Sarah Kuba, Thomas Ruppel, Katrin Balzer, Sascha Köpke, Indre Maurer, Clarissa E. Weber, Martin Scherer, Eva Hummers, Christiane A. Müller

**Affiliations:** 1 Department of General Practice and Primary Care, University Medical Center Hamburg-Eppendorf, Martinistraße 52, Hamburg, 20246 Germany; 2 Department of General Practice, University Medical Center Göttingen, Humboldtallee 38, Göttingen, 37073 Germany; 3 Institute for Social Medicine and Epidemiology, Nursing Research Unit, University of Lübeck, Ratzeburger Allee 160, Haus 50, Lübeck, 23538 Germany; 4 Institute of Nursing Science, University of Cologne, Medical Faculty & University Hospital Cologne, Gleueler Straße 176-178, Cologne, 50935 Germany; 5 Rechtsanwaltsgesellschaft Dr. Ruppel mbH (law firm for medical law), Moislinger Allee 9d, Lübeck, 23558 Germany; 6 Chair of Organization and Corporate Development, Faculty of Business and Economics, Georg-August-University Göttingen, Platz der Göttinger Sieben 3, Göttingen, 37073 Germany; 7 Department of Business and Society, School of Business and Management, Queen Mary University of London, Bancroft Building, Mile End Campus, Mile End Road, London, E1 4NS UK; 8 School of Public Health, Health Services Research and Nursing Science, Bielefeld University, Universitätsstraße 25, 33615 Bielefeld, Germany; 9 Department of Nursing and Health Care, Hochschule Hannover University of Applied Sciences and Arts, Blumhardtstraße 2, Hannover, 30625 Germany

**Keywords:** Outpatients, Nursing care, Home care, Interprofessional relations, Patient-centred care

## Abstract

**Background:**

People receiving home care (PRHC) usually have complex healthcare needs that require the involvement of informal caregivers and health professionals. Interprofessional collaboration is an important element of person-centred home care, which is often insufficiently implemented. This study aims to explore current practices of collaboration in the home care setting and develop an interprofessional person-centered care concept.

**Methods:**

The care concept was developed iteratively by incorporating theory and evidence and involving interest holders. The evidence was synthesized from a literature review, semistructured interviews with PRHC (*n* = 20) and relatives (*n* = 21), three mono-professional focus groups each with nurses (*n* = 17), general practitioners (*n* = 14) and therapists (*n* = 21). The findings were discussed in four mixed focus groups (*n* = 37). Qualitative data were analysed using content analysis. In addition, a survey involving PRHC and relatives (*n* = 37), nurses (*n* = 70), general practitioners (*n* = 33) and therapists (*n* = 81), and eight best-practice cases were explored based on nine home visit observations, 29 interviews and shadowings using principles of grounded theory. The quantitative data were analysed descriptively. Finally, the care concept was outlined in an expert workshop (*n* = 25) and finalised in a co-creation workshop (*n* = 12).

**Results:**

The developed interprofessional, person-centered care concept for PRHC includes five components: the designation care coordinators, an initial joint home visit, a secure digital communication system, a dedicated phone number for quick contact, and additional joint meetings as needed. The approach highlights individualized, goal-oriented care plans, sustained and effective interprofessional collaboration, and active engagement of PRHC and their relatives in refining and delivering care in accordance with their own goals and needs. An accompanying logic model supports systematic guidance for planning, implementation, and evaluation, enhancing the likelihood of success and ensuring a transparent, well-structured process.

**Conclusions:**

Interprofessional, person-centred approaches to home care in Germany should focus on improving communication, shared responsibility and joint care planning between PRHC, relatives and healthcare professionals. The aim of the developed care concept is to improve quality of life and reduce hospital admissions of PRHC. This concept will first be evaluated and refined in a cluster randomized controlled trial.

**Trial registration:**

This study is registered on ClinicalTrials.gov as NCT05149937 on 03/11/2021. Study part A completed.

**Supplementary Information:**

The online version contains supplementary material available at 10.1186/s12875-025-03098-0.

## Background

The number of People Receiving Home Care (PRHC) is expected to rise in the coming decades [1]. Home care aims to meet the health and social needs of PRHC by providing high-quality healthcare and social services through formal and informal caregivers and using technology where appropriate [1]. As home care involves a variety of people, such as relatives, nurses, general practitioners (GPs), occupational therapists, speech therapists and physiotherapists, among others, constant mutual coordination is needed. This is rarely done in a systematic and structured manner [2]. The World Health Organization (WHO) defines ‘collaborative healthcare practice’ as follows: “… when multiple healthcare professionals from different professional backgrounds provide comprehensive services by working with the person herself/himself, relatives, providers and communities to deliver the highest quality of care across settings” [3]. Inadequate collaboration and communication between the healthcare professionals involved can lead to “inconsistent care” due to problems in sharing information [2]. This has been shown to have a negative effect on the safety of those in need of care as a result of errors, for example, in connection with medication [4]. In addition, client-reported coordination problems are associated with unscheduled urgent medical visits and hospitalizations. Successful care coordination is associated with higher levels of care quality from the staff or the client´s perspective [5]. Person-centred care is an approach to achieve improvements in safety, quality, and coordination of healthcare as well as in quality of life for older people with multiple chronic conditions and/or functional limitations. In person-centred care, the individual’s values and preferences guide all aspects of healthcare in a dynamic relationship between the individual, significant others, and all relevant healthcare providers [6]. To date, studies in the home care setting have focused exclusively on collaboration between only two professional groups and often have not included the PRHC and relatives. Existing evidence generally relates to integrated or coordinated care approaches for people with specific chronic conditions or to palliative care. Research into routine care for PRHC has been lacking.

When it comes to collaboration between GPs and nurses of German outpatient nursing services, the latter rate communication and documentation in the context of collaboration as irregular, cumbersome and unsatisfactory [2] and issuing prescriptions as conflictual [7]. Mutual respect [5, 8], a fixed contact person and additional remuneration for collaboration [5], e.g., for participation in structured team meetings [8], as well as defined structures and common goals [9], are crucial factors for establishing and maintaining effective collaboration between physicians and nurses in primary care.

Occupational therapists and physiotherapists feel that communication with GPs, outpatient care services and other therapists regarding joint patients takes place too rarely [10]. Physiotherapists and GPs both perceive mutual communication and the receipt of appropriate examination findings as important [11]. Closer collaboration can lead to better management of patients with complex problems and prevent inappropriate referrals [12].

Although practice-based interprofessional collaboration interventions can improve healthcare processes and outcomes [13], only a few structured approaches have been implemented in the home care setting thus far. Concepts such as “home-based primary care teams” in Canada [14] cannot be transferred to the German healthcare system. In Germany, service providers do not work within a team but rather as individual providers who offer and bill their services independently with statutory or private health insurance. Moreover, patients are generally free to choose their health care providers. Current projects in Germany are attempting to improve collaboration between GPs and nursing staff, for example, by developing regional network forms of organization, implementing case and care management in the provision of care for elderly patients [15–17] or using telehealth platforms [18] to optimize outpatient care. However, none of these projects aims to directly promote interprofessional collaboration, including also occupational therapists, physiotherapists and/or speech therapists. Additionally, the implementation of person-centred care has not been a priority thus far. “Person-centered care” is defined as an approach that emphasizes the individual needs, preferences, and values of older adults in the delivery of healthcare services. One key element is fostering strong partnerships between healthcare providers and patients. This involves effective communication, sharing of information, and collaboration in decision-making [6]. For these reasons, our study was conducted using a participatory approach in which relevant groups involved in home care actively contribute to the development of the interprofessional person-centred concept.

The overall aim of this study was to develop an interprofessional person-centred care concept for PRHC, based on an exploration of the current practices of independent health care professionals in four German citys and their regions. This concept systematically considers the perspectives of PRHC, relatives, nurses of outpatient nursing services, GPs, occupational therapists, physiotherapists and speech therapists.

## Methods

### Study design

We adhered to the GUIDED guideline for reporting for intervention development studies when describing the development process in detail [10].

**Fig. 1:**
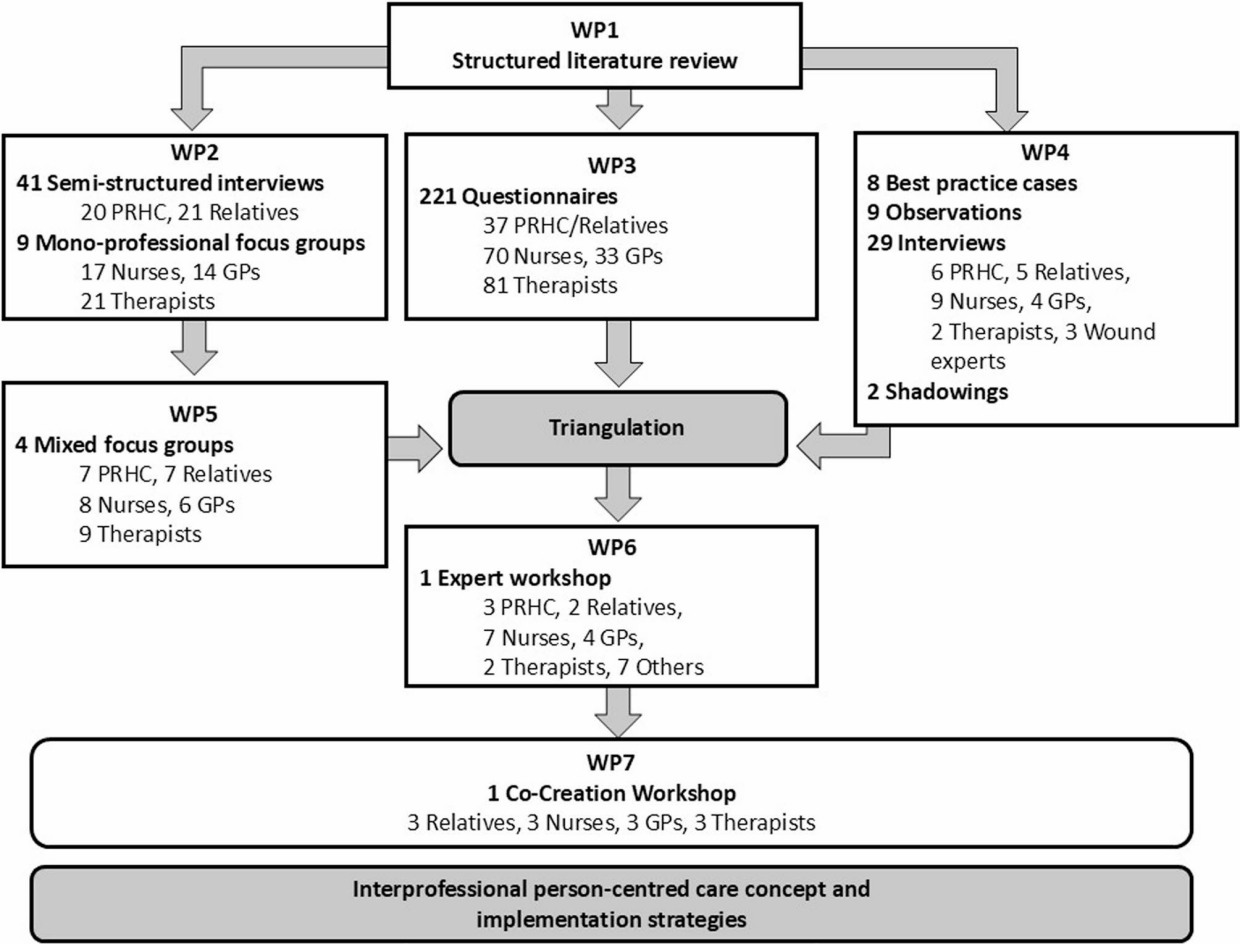
Study design for the *interprof* HOME development, WP = work package

The interprofessional person-centred care concept *interprof* HOME was developed according to the MRC framework for the development and evaluation of complex interventions [11] using a multistep mixed methods approach. This paper describes the development phase, where we consider the core elements of the MRC framework: considering context, developing and refining program theory, engaging interest holders, identifying key uncertainties, refining the intervention, and economic considerations [11]. The development process consisted of six work packages (WP1-6) that were carried out simultaneously or consecutively (Fig. 1), as described in detail in our study protocol [12]. In addition, we conducted a co-creation workshop (WP7). The study took place between May 2021 and September 2023, and the co-creation workshop occurred in October 2024.

### Study setting

The study was conducted in the city and surroundings of Göttingen, Hamburg, Lübeck and Cologne. The researchers of the respective research teams had various areas of professional expertise and experience in general practice, nursing practice, nursing pedagogy and nursing research, public health, organizational and corporate development, medical law, physiotherapy, or occupational therapy. In addition, the study team was supported by a multiprofessional advisory board made up of patient representatives, members of self-help groups and professional carers.

### Participants and sampling

Recruitment of participants for interviews (WP2), focus groups (WP2 and WP5), best-practice cases, observations, shadowings (all WP4) and co-creation workshop followed the same procedure: professionals were identified using local registers and invited to participate in the study by letter and later by telephone. The PRHC and relatives were recruited through outpatient nursing services, self-help groups, GPs and therapists. Additionally, professionals identified from local registers were invited to participate in the survey via email, followed by two reminders (WP3). The PRHC and relatives were recruited using notices in care support centres, information in newsletters or websites of caregiving relatives or self-help groups and presentations of the study in meetings of self-help groups. The experts for the workshop (WP6) were invited on the basis of their experiences, the recommendations of the advisory board or professional contact. The researchers contacted them via email or telephone. The eligibility criteria for participants are described in our study protocol [12]. For the co-creation workshop (WP7) patients, relatives, registered nurses, GPs and therapists were invited to discuss the components of the *interprof* HOME concept after WP6. They were purposefully recruited with regard to heterogeneous characteristics (gender, age, and residence).

### Data collection and analysis

#### Step 1: identifying existing evidence and theory

Initially, existing evidence was synthesized by carrying out two literature reviews.

##### Literature review (WP1)

First, a literature review of professional providers’, PRHC’, and relatives’ perspectives on collaboration and communication over the previous 10 years was conducted. The research questions were: (1) What is the perspective of general practitioners/professional caregivers from outpatient care services/therapists on cooperation/communication with other caregivers? (2) What is the perspective of PRHC/relatives on cooperation/communication with professional caregivers? We conducted systematic searches in the Cochrane Library, PubMed, EMBASE, CINAHL, and PsycINFO for studies published in English or German. The search was performed by different members of the research team, with five separate foci: general practitioners, nursing staff, therapists, relatives, and PRHC. For all searches, we applied a core set of MeSH terms (*“Interpersonal Relations”*, *“General Practitioners”*, *“Home Care Services”*, *“Nurses”*, *“Occupational Therapists”*, *“Physical Therapists”*, *“Patients”*, *“Family”*). Search term combinations were adapted according to the respective perspective.

In a second literature review, intervention studies on strategies for promoting interprofessional collaboration in PRHC outpatient care were reviewed. Systematic searches using sensitive search strategies were carried out in the databases MEDLINE (via PubMed), Cochrane Library, CINAHL and PEDro, coupled with manual searches in citations lists of relevant articles. A primary search strategy was initially developed for the MEDLINE database combining major subject headings and freetext vocabulary on the concepts ‘outpatient care’ and ‘interprofessional collaboration’; this strategy was subsequently adapted to the other databases.

#### Step 2: exploring the perspective of all involved groups

To develop a customised and successful care concept, we used the participatory approach by considering the perspectives of all the groups involved and working with them to enable the inclusion of their different needs and perspectives.

##### Qualitative interviews and mono-professional focus groups (WP2)

The interprofessional healthcare situation of the PRHC was explored via semistructured interviews (facilitators: BT, RD, CH, AM, US, TH, and LS) with the PRHC and relatives. After obtaining informed consent, trained members of the research team conducted open guideline interviews in face-to-face, online or telephone interviews The results from WP 1 served as the basis for developing the interview guides for WP2. For guidelines for interviews with PRHC and relatives see Supplementary Material 1 in [19]. Monoprofessional focus groups, either with GPs, nurses from outpatient nursing services or therapists, were conducted in the four study centres using video conferences (facilitators: BT, RD, CH, AM, US, TH, AK). For interview guideline for monoprofessional focus groups with nurses, GPs and therapists see Supplementary Material 2 in [19]. The barriers to and facilitators of interprofessional person-centred care were discussed, and first, ideas for improvement were collected.

The interviews and focus groups were audio recorded, transcribed and checked against the audio recording, and the identifiers of persons and locations were replaced with pseudonyms. The interviews and focus groups were analysed per person group using qualitative content analyses in teams of at least two researchers from different centres [20]. 

##### Survey (WP3)

In a multicentre survey, PRHC/relatives, GPs, nurses and therapists completed questionnaires on the following four topics: (1) previous cooperation in home care, (2) possibilities of cooperation, (3) starting points for cooperation, and (4) ideas for measures to promote interprofessional cooperation. In order to take into account the perspectives of all those involved in the care of PRHC, we created four different questionnaires. Data were collected anonymously via the “SoSci Survey” online platform [21] and analysed descriptively using the IBM SPSS Statistics for Windows software, version 28.0.

##### Best-practice cases (WP4)

Data on eight home-care constellations considered “best-practice cases”, that is, cases in which all involved actors agreed that collaboration was working particularly well, were collected through non-participatory observation of professionals’ home visits and semi-structured interviews with the PRHC, relatives or close contacts, nurses, GPs, therapists and wound experts (facilitator: MD). To select best-practice cases, we asked professionals (GPs, nurses, therapists) to identify a patient case they perceived as exemplary in terms of collaboration. They then asked the other actors involved (patients, relatives, other professionals) for their consent to participate. If all parties agreed and confirmed the high quality of collaboration, the case was included as a best-practice example. The guiding themes in interview were (1) general overview of the care and collaboration taking place in this specific case, (2) coordination and information exchange between actors involved, (3) home visits of professional actors, (4) space (role/use of living space for care and collaboration), (5) problems, (6) changes in conditions/hospitalization and (7) general idea of (interprofessional and professional/non-professional) collaboration. For the professional actors we also included: (8) differences of this case compared to other cases. The interviews were audio recorded, transcribed and pseudomized to enable a detailed analysis of the guiding themes. During the observations, notes were taken. During preparation and follow-up of observations, informal conversations were held with the different professional and non-professional actors. In addition, two nurses from outpatient nursing services were shadowed during the workday (18 hours in total) to gain insights into the organization of their daily routines, and notes were taken. A case-based analysis based on principles of grounded theory was conducted for the evaluation proceeding from individual case analyses to a systematic cross-case comparison [22, 23]. We focused on the practices and measures of the individual actors that fostered collaboration and then compared these across the eight cases.

##### Mixed focus groups (WP5)

Mixed focus groups (facilitators: BT, RD, CH, AM, US, and TH) with samples of representatives from all parties were involved to outline the components of the interprofessional person-centred care concept. The findings of WP1 to WP4 were used as a basis for developing the guideline for the mixed focus groups with the following topics: A: presentation and discussion of barriers and facilitators to collaboration: (1) availability of relatives and professionals, (2) care partnership between relatives and professionals, (3) lack of established processes: fixed contact person, shared (digital) documentation, and communication system and (4) being known and trust; B: presentation and discussion of wishes/ideas for improved collaboration: (1) reducing the burden on patients and relatives regarding care organization and coordination, (2) contact information and appointment coordination, (3) (digital) documentation and communication tools, (4) personal (direct) exchange, (5) interprofessional education and training, (6) fixed contact persons and/or care teams. C: discussion of future interprofessional collaboration in PRHC care: (1) feasibility, (2) implementation strategies, (3) involved actors. The first two topics focus on the content reported in the WPs. The third topic focuses on the needs and preferences of PRHC and relatives in order to ensure person-centred, high-quality healthcare at home. Focus groups were audio recorded, transcribed and checked against the audio record, and the identifiers of persons and locations were replaced with pseudonyms. Analyses were performed using content analyses with a focus of topics and subtopics in the analysis process [20].

In a second step we used joint displays [24] with regard to the identified categories focusing on the areas of action in which interprofessional cooperation needs to be improved, we were able to compare the results of WP5 simultaneously and in relation to the results of the interviews and focus groups (WP2), of the survey (WP3) and best-practice cases (WP4) in a tabular presentation and write a summary of the content. The findings, containing a preliminary list of potential components of the care concept (areas of action), were sent to the experts of the workshop (WP6) in advance.

#### Step 3: modelling the first version of the concept and implementation strategies

##### Expert workshop (WP6)

During an online expert workshop, the participants discussed, adapted and combined the components of the care concept that had been drafted. The experts were allocated to four break-out groups focusing on different topics which the preliminary areas of action were assigned to: (1) expansion of the scope of action for the nursing staff and therapists and continuing education to increase competencies in nursing and in medical and therapeutic activities for the professional groups involved in home care as well as for relatives; (2) coordination and planning of joint home visits; (3) communication by telephone, face-to-face communication, documentation and coordination of appointments; and (4) digital communication/documentation systems. Each group assembled a mix of professional and PRHC/relatives perspectives. Within these group sessions, the participants reflected on the relevance and feasibility of these areas and discussed, based on their experiences and presented results, specific measures that should be implemented. For each measure they discussed the aims, involved parties and their roles, occasions and indications for implementation, potential impact on trust-building between PRHC, relatives and professionals, specific wishes and needs of PRHC and implementation considerations. One facilitator and one person taking minutes were present in each digital (breakout) room. The results of the break-out groups were discussed in a subsequent plenary session, leading to a preliminary list of intervention components and associated measures. In a subsequent online survey during the meeting, the participants assessed the relevance and feasibility of these measures. After the results of this survey were discussed, the measures were prioritized by selection of those that were most frequently chosen by the participants in a second live online poll. In this poll, that closed the workshop discussion on the intervention components, each participant could choose up to three measures (one in the area of care coordination, two in the other areas). Thereafter relevant factors for implementation were discussed in four groups via SWOT (Strengths, Weaknesses, Opportunities, Threats) analysis. Additionally, relevant context factors across measures were identified. Finally, implementation strategies were derived and briefly discussed using the CFIR-ERIC strategy matching tool [25, 26]. The expert workshop was conducted over two half-days and was logged and documented.

#### Step 4: first version of the interprofessional person-centred care concept

The research team refined the components of the care concept through several meetings, considering the findings across all WPs with the main emphasis on the expert workshop. The components of the new interprofessional care concept and the implementation strategy were documented in an intervention protocol according to TIDieR [27] (Additional file 1).

#### Step 5: modelling the final version of the concept and implementation strategies

##### Co-creation workshop (WP7)

As the first version of the *interprof* HOME concept was refined by the research team from the collection of topics from the expert workshop, its suitability for daily use in routine care needed to be proven. We conducted a co-creation workshop in order to explore the relevance, feasibility and robustness of the components. Therefore, a two-day co-creation workshop was held in person at the Department of General Practice in Göttingen, each lasting 4 hours [28, 29]. Here, implementation of the components was simulated against real-life care scenarios. In advance, the first version of the care concept *interprof* HOME was sent to the participants via email. On the first day, the participants were divided into two subgroups. Each subgroup consisted of individuals from different groups (relatives, registered nurses, GPs, and therapists) to ensure constructive multi-perspective exchange. During the sessions, both subgroups adapted half of the components according to different care scenarios in a simulation game. The findings were documented by mapping methods on both days. On the second day, the interim adapted *interprof* HOME concept was briefly presented to the (partially new) participants. Now, the two subgroups adapted half of the components again, according to the other care scenario, in a simulation game. In a common session, the subgroup results were analysed, and the new components were consented. These components were sent to the participants for additional comments. Afterwards, the concept was finalized by the research team.

## Results

### Literature review (WP1)

In the first literature review, 28 studies on the topic of collaboration and communication were included. In terms of study design, most of these were qualitative studies, some were quantitative, and a few had different designs (Table 1). Most of the studies focussed on GP’s, nurses´ and/or PRHC’ perspectives. None of the studies dealt exclusively with the perspective of therapists (Table 2). A realist review focussed on the complex mechanisms underlying team-based delivery of person- and family-centred home care [30].

**Table 1:** Study design of the studies included in the structured literature review (*n* = 28)

Design	Number	Source
Qualitative	19	[32–50]
Quantitative	5	[51–55]
Mixed method	1	[56]
Scoping review	1	[31]
Realist review	1	[30]
Case study	1	[57]

**Table 2:** Perspectives of the studies included in the structured literature review (*n* = 27)

Perspectives	Number	Source
PRHC	4	[31, 53, 56, 57]
Relatives	3	[33, 40, 42]
PRHC and relatives	3	[32, 37, 50]
Nurses	5	[46–48, 51, 54]
GPs	2	[34, 52]
Nurses and GPs	3	[35, 36, 45]
PRHC, nurses and GPs	1	[55]
Nurses and relatives	1	[39]
GP and PT	1	[38]
Nurses, OT and PT	1	[43]
Nurses, physicians and therapists	3	[41, 44, 49]

*PRHC* people receiving home care, *GPs* general practitioners, *OT* occupational therapists, *PT* physiotherapists

One scoping review [31] and one qualitative study [32] focused on the home care of patients with Parkinson’s disease, three studies focused on palliative home care [9, 33, 34], and two focused on collaboration in patients’ transitions from hospital to home [35, 36].

The main findings from the first literature review show that relatives see themselves not only as coproviders but also as corecipients [41]. Patients and relatives in palliative care at home expect typically good availability of their GP and care professionals to work together well and transparently, including through joint documentation [37]. Nurses see themselves as a central and coordinating unit [43] but would like to have more attention and trust from GPs [44]. The participation of GPs in teams depends on their personal decisions [49].

The second literature review revealed that strategies that promote interprofessional collaboration in home care included initial assessment of the patient’s state of health; their needs, wishes and goals; home visits; care planning; and interprofessional exchange and coordination [58–60] through availability by telephone [60] or a web-based communication tool [61]. None of the studies included therapists.

### Qualitative interviews and mono-professional focus groups (WP2)

Semi structured interviews were conducted with a total of 20 PRHC and 21 relatives. The interviews lasted 20–76 min (PRHC) and 16–89 min (relatives). The group size of the 9 mono-professional focus groups varied between 4 and 7 participants. Overall, 17 nurses, 14 GPs and 21 therapists (8 occupational therapists, 6 physiotherapists and 7 speech therapists) participated.

The recommendations for better collaboration and communication extracted from the interviews and the mono-professional focus groups resulted in the following three main categories: (1) perception of interprofessional collaboration, (2) means of communication and (3) barriers and facilitators. The participants’ characteristics and detailed findings have been published in more detail elsewhere [19].

### Survey (WP3)

In the survey, 37 PRHC/relatives, 33 GPs, 70 nurses and 81 therapists completed the questionnaire. More than half of the PRHC/relatives indicated dissatisfaction with collaboration and agreements between professional groups and felt that professional groups did not serve their best interests (46% “dissatisfied”, 8% “very dissatisfied”). Nurses, GPs and therapists assessed interprofessional communication among themselves as a very important part of their nursing, medical and therapeutic responsibilities (nurses: 55% “strongly agree”; GPs: 54% “strongly agree”; TH: 48% “strongly agree”). They advocated using measures such as fixed telephone/video consultation hours and joint home visits to strengthen this communication. However, they were sceptical regarding the implementation of these measures due to a lack of time and human resources. The findings will be published in more detail soon.

### Best-practice cases (WP 4)

A total of 8 “best-practice cases” were analysed on the basis of 9 observations of home visits, 29 interviews and 2 shadowings. We found that in these best-practice cases, both the PRHC and/or relatives often played a major role in the coordination of the (inter-)professional work, supporting the notion that coordination and the role of a dedicated coordinator is crucial. PRHC or relatives either actively orchestrated connections between the professionals to facilitate their own coordination. Alternatively, they continuously coordinated the procedures and activities across professionals on an ongoing basis themselves. The findings will soon be published in more detail elsewhere.

### Mixed focus groups (WP5)

The four mixed focus groups included 7 PRHC, 7 relatives, 8 nurses, 6 GPs and 9 therapists (4 occupational therapists, 2 physiotherapists and 3 speech therapists). The group size varied from 8 to 10 participants. The participants’ characteristics are presented in Table 3. A total of twelve areas of action where the interprofessional collaboration needed to be improved were identified in the focus groups: (1) expansion of the scope of independent action for nurses and therapists by the use of blank prescriptions and pro re nata medication, (2) continuing education for the professional groups involved in home care as well as for relatives, (3) a contact person/coordinating person, and (4) paper-based communication/documentation systems (this means that a standardized procedure should be established with regard to communication/documentation systems as contrasted with, e.g., communication & documentation by email, such as a documentation folder that is shared in the home care setting or via a digital platform), (5) digital communication/documentation systems, (6) digital appointment coordination, (7) paper-based appointment coordination, (8) communication and documentation by email, (9) communication and documentation by fax, (10) communication by telephone, (11) planned joint home visits and (12) planned (regular) meetings. An additional focus was placed on the improvement of person-centredness: the inclusion of wishes, needs and resources of the PRHC and relatives, as well as the promotion of trust and mutual familiarity among the actors.

**Table 3:** Characteristics of the participants (mixed-professional focus groups)

Variables	PRHC (*n* = 7)	Relatives (*n* = 7)	Nurses (*n* = 8)	GPs (*n* = 6)	Therapists (*n* = 9)
**Age in years**,** min–max** Mean Standard deviation Median	38–64 51.57 9.93 54.00	45–74 57.86 9.94 57.00	38–60 ^d^ 47.71 9.25 42.00	37–57 46.00 7.75 43.00	25–47 37.22 8.24 36.00
**Female**,** n (%)**	3 (42.9%)	4 (57.1%)	7 (87.5%)	4 (66.7%)	7 (77.8%)
**City size, n (%)**					
< 5.000 inhab. 5.000–20.000 inhab. 20.001-100.000 inhab. > 100.000 inhab. Missing	4 (57.1%) - 1 (14.3%) 2 (28.6%) -	1 (14.3%) 3 (42.9%) - 3 (42.9%) -	3 (37.5%) 1 (12.5%) 1 (12.5%) 3 (37.5%) -	2 (33.3%) 3 (50.0%) - 1 (16.7%) -	- 3 (33.3%) 2 (22.2%) 4 (44.4%) -
**Level of care dependency**,** n (%)**^**a b**^					
0 1 2 3 4 5 Missing	- - - 3 (42.9%) 2 (28.6%) 1 (14.3%) 1 (14.3%)	- - 3 (42.9%) 2 (28.6%) 1 (14.3%) 1 (14.3%) -			
**Duration of care dependency**,** n (%)**					
1–5 years 6–20 years 21–35 years Missing	4 (57.1%) 2 (28.6%) - 1 (14.3%)	5 (71.4%) 2 (28.6%) - -			
**Prescription for…**,** n (%)**^**ac**^					
Occupational therapy Physiotherapy Speech therapy	3 (42.9%) 7 (100.0%) 1 (14.3%)	2 (28.6%) 2 (28.6%) -			
**Years of work experience**					
**Min–max** Mean Standard deviation			12–42 25.88 10.32	9–30 17.00 8.85	1–21 11.64 8.18
**Years of work experience, n (%)**					
1–5 years 6–20 years 21–35 years > 36 years Missing			- 3 (37.5%) 3 (37.5%) 2 (25.0%) -	- 4 (66.6%) 2 (33.3%) - -	3 (33.3%) 4 (44.4%) 1 (11.1%) - -

*PRHC* people receiving home care, *GPs* general practitioners, *inhab* inhabitants

^a^The data refers to the respective PRHC

^b^Long-term care grade: 0 = no impairment of independence or capabilities; 1 = low level of impairment of independence or capabilities; 2 = significant level of impairment of independence or capabilities; 3 = serious level of impairment of independence or capabilities; 4 = the most severe level of impairment of independence or capabilities; 5 = the most severe level of impairment of independence or capabilities with special long-term care requirements

^c^Multiple answers were possible

^d^One answer was missing

### Expert workshop (WP6)

The results of the previous steps were made available in advance to the participants of the expert workshop. A total of 25 experts (3 PRHC, 2 relatives, 7 nurses, 4 GPs, 2 therapists, 2 representatives of state healthcare organizations, and 1 person each from the self-help groups, the field of medical law, medical professionals, telemedicine and social service providers) participated. The triangulated findings were grouped in four topic areas: (1) expansion of the scope of action for the nursing staff and therapists and continuing education to increase competence in nursing and in medical and therapeutic activities for the professional groups involved in home care as well as for relatives; (2) coordination and planning of joint home visits; (3) communication by telephone, face-to-face communication, documentation and coordination of appointments; and (4) digital communication/documentation systems and coordination of appointments and video conferences for case discussions. Discussions of these four topic-specific breakout groups yielded a preliminary list of 15 specific measures related to the areas of action identified in the previous study parts. These measures were rated and discussed by the participants with regard to relevance and feasibility. With these shared views and opinions in mind, the participants finally voted for their individual top 3-measures to be implemented. Results of this voting led to the following list of prioritized measures to improve interprofessional collaboration and person-centred care were identified: (1) assignment of the coordinating role to the person with care needs or their caregiving relatives, (2) promotion of interprofessional collaboration at the regional level, (3) a telephone number for use by all the parties involved in jointly pre-defined situations, (4) a digital communication system, (5) regular joint home visits with all the persons involved, and (6) event-related/regular online meetings of the professionals involved with the PRHC and/or relatives. Measures that were rated with lower priority and thus not selected for the intervention to be implemented were related to care coordination by extra case managers or by other à priori defined professionals, shared patient documentation, or activities for continuing interprofessional education. In the SWOT analysis factors supporting implementation, such as mutual familiarity and trust among the professionals involved, the PRHC and their caregivers, were discussed and regarded as beneficial for interprofessional care. Financial incentives were also perceived as supportive. The participants noted that data protection regulations had to be monitored and considered in the area of communication systems. The PRHC expressed concerns that participation in digital communication/online meetings might become too strenuous for them.

The following relevant implementation strategies were ultimately derived using the CFIR-ERIC strategy matching tool [25]:

- Identify and prepare champions.
- Assess for readiness and identify barriers and facilitators.
- Capture and share local knowledge.
- Inform local opinion leaders.
- Alter incentive/allowance structures.
- Access new funding.
- Build a coalition.
- Obtain and use patients/consumers and family feedback.
- Conduct educational outreach visits.
- Promote adaptability of the intervention measures.

The implementation strategies were not discussed in more detail with regard to specific implementation measures to be used in the project.

### First version of the interprofessional person-centred care concept

The first version of the person-centred care concept was defined by the research team on the basis of the results of the expert workshop and findings from the other WPs. The results from the discussions with the advisory board with regard to the logic model and the care concept were also taken into account. The first version of the interprofessional person-centred care concept contained the following six components:

- *Kick-off meeting*: All the persons (including the PRHC and, if applicable, their relatives) involved in the care process meet to get to know each other. The tasks in the project are explained, responsibilities are established, and upcoming meetings are scheduled.
- *Designation and support of a coordinator*: The coordinator is in charge of supporting the PRHC and, if necessary, a relative by organizing coordination across all actors involved.
- *Digital communication system (messenger)*: A digital communication system is set up for the scheduling of meetings as well as a quick exchange of information.
- *Designated telephone number*: All the professional caregivers set up a special telephone number to be reachable in more urgent incidents. Purposes and times of use are discussed by the interprofessional team.
- *Joint home visit*: A joint home visit is conducted up to 3 months after the kick-off meeting to discussthe current concerns of the PRHC and how to proceed are discussed is conducted up to 2 months after the kick-off meeting.
- *Joint meetings*: Here, the health-related concerns of the PRHC are re-evaluated and care is adapted. The first joint meeting takes place up to three months after the joint home visit. Thereafter, joint meetings will be held on an as-needed basis.

### Co-creation workshop (WP7)

A total of 12 people attended one or both days of the workshop. On the first day, 10 participants (2 relatives, 3 nurses, 3 GPs, and 2 therapists), and on the second day, 9 participants (2 relatives, 2 nurses, 2 GPs, and 3 therapists) took part. Seven participants attended both days of the workshop. The participants’ characteristics are presented in Table 4.

**Table 4:** Characteristics of participants (co-creation workshop)

Variables	Relatives (*n* = 3)	Nurses (*n* = 3)	GPs (*n* = 3)	Therapists (*n* = 3)	All (*n* = 12)
Age in years, min–max Mean Median	51–84 65.3 61	49–61 55.3 56	41–52 46.3 46	25–34 31 34	25–84 50.1 51
Female, n (%)	3 (100%)	2 (66.7%)	1 (33.3%)	1 (33.3%)	7 (58.3%)
City size, n (%)					
< 5.000 inhab. 5.000–20.000 inhab. 20.001-100.000 inhab. > 100.000 inhab. Missing	- 2 (66.7%) 1 (33.3%) - -	- 2 (66.7%) 1 (33.3%) - -	- 1 (33.3%) 2 (66.7%) - -	- 1 (33.3%) 2 (66.7%) - -	- 7 (58.3%) 5 (41.7%) - -
Duration of care dependency, n (%)					
> 5 years 6–20 years 21–35 years Missing	2 (66.7%) - - 1 (33.3%)				
Years of work experience					
Min–max Mean		23–45 35.3	2–10 5.3	1–11 6.7	

*GPs* General practitioners

The co-creation workshop took place as planned. The participants valued the existing concept but proposed several refinements. During the subgroup and the plenum sessions, the components of the *interprof* HOME concept were slightly adjusted and aggregated to facilitate its implementation in routine care. The relevance of the previously developed intervention components was largely confirmed. The few modifications included: (1) specifying that nurses, together with another caregiver, act as coordinators (“interprof HOME agents”); (2) reducing the number of obligatory joint home visits to one (instead of a kick-off meeting plus a joint visit); and (3) further joint home visits to take place solely on an as-needed basis. The final interprofessional person-centred care concept for PRHC involves the following five components:

- *Designation of coordinators*: One nurse providing routine care counselling to the PRHC training will be appointed to act as a specialized *interprof* HOME care coordinator (*interprof* HOME agent). The *interprof* HOME agent and additionally a relative (or another person involved in care) share the coordinating tasks according to an agreement before the initial joint home visit. The coordinators are responsible for supporting the PRHC and their relatives.
- *Initial joint home visit*: An initial joint home visit is carried out by all involved health professionals and, if possible, a relative to (1) introduce the *interprof* HOME concept (to clarify the tasks and responsibilities of each person in this context and to schedule upcoming meetings if necessary) and (2) develop an individual goal-oriented interprofessional treatment plan. The initial joint home visit can be initiated by any person of the interprofessional team – including the PRHC themselves. In advance, the *interprof* HOME agent (see previous point) collects the needs of the PRHC. The visit is organized, facilitated, and minuted by the *interprof* HOME agent. This approach contains the following person-centred elements: the treatment plan is based on the PRHC’s preferences, and the PRHC explicitly considered as an integral member of the interprofessional team. Moreover, the participants are introduced in the principles of person-centred care to support constantly the PRHC’s autonomy.
- *Digital communication system (messenger)*: A data protection-compliant digital communication system is set up during the two weeks following the initial joint home visit for a quick exchange of information, photos, or short videoclips. It can also be used to schedule the joint home visit and joint meetings; according to the software, video conferences may also be possible. The PRHC decides, to which extend he/she wants to be included in the communication of health professionals another element of person-centred care.
- *Designated telephone number*: All professional caregivers set up a special mobile/telephone number to be available directly to other persons during fixed times. The aim is to ensure faster accessibility and improved communication in more urgent incidents (not in emergencies! ). In line with person-centred care, also the PRHC has the option to contact professional caregivers directly per telephone or via the *interprof* HOME agent.
- *Joint meetings if necessary*: In further joint meetings, the health-related concerns of the PRHC are re-evaluated, and further actions are discussed. They take place online/in the practices of involved professionals or in the home care setting and can be initiated by all individuals in the care of the PRHC if needed. Again, the needs of the PRHC will be collected in advance by the *interprof* HOME agent. The intervention protocol (Additional file 1) according to TIDieR [27] displays the final version.

A preliminary logic model (Additional file 2) was developed on the basis of the outcomes in WP6. It links the intervention components to relevant change mechanisms and target outcomes, taking into account potential facilitators for the implementation of the components. Currently, it is mainly a theoretical model; its implementation and sustainability should be tested in practice regarding PRHC-related outcomes, accompagnied by a process evaluation in line with the the MRC framework [11].The preliminary logic model will be further refined into a version suitable for routine care. By applying the final logic model, interest groups and implementers can enhance the planning, implementation and evaluation of *interprof* HOME, thereby increasing the likelihood of success. The logic model also facilitates transparent presentations of the desired outcomes and underlying assumptions, while serving as a training support for stakeholders.

## Discussion

In this article, we described the development of the complex intervention “*interprof* HOME - an interprofessional person-centred care concept for persons with care needs living in their own homes.” The care concept comprises five components.

According to our findings, communication both between PRHC/relatives and professionals and in interprofessional teamwork is seen as key to success. Interviewed PRHC, their relatives and participants of the monoprofessional focus groups explicitly stated that direct exchanges between professionals who know each other result in intensive exchange and closer working relationships that optimize healthcare. Also participants of the online survey considered interprofessional communication as an important aspect in their caring responsibilities. In the current situation in Germany, professionals caring for one PRHC even often do not know each other, which is why meetings implying communication between participants are needed to establish structures for collaboration. Currently also no joint planning of care requirements takes place in PRHC care. To improve communication, the participants of our mixed focus groups, expert workshop and co-creation workshop were in favour of measures such as fixed telephone/video consultations and joint home visits. In addition, it was demanded that both, the PRHC and their relatives need to be supported. In particular, when a relative (PRHC) requires support, informal caregivers are often overwhelmed by the abundance of new and unknown tasks. Moreover, they do not want to delegate all these tasks to a third party. In line with our findings, other projects also reported that intensive communication (clear agreements, openness, transparency) [54], training and workshops for knowledge sharing [62], joint home visits [36], regular team meetings [46], and simpler structures [54] may help improve interprofessional home healthcare. For example, transparency could be improved through joint (digital) documentation [47].

*Interprof* HOME and its findings are unique in Germany. Other projects have described only barriers and facilitators but have not offered a concept for improving the current situation. International studies have also focussed more on the type and nature of cooperation, particularly in palliative care, than on the development or testing of new concepts [63]. Our care concept addresses the importance of the quality of personal relationships with collaboration partners to improve collaboration [53]. It also addresses the essential elements for home care from the perspective of the PRHC and relatives, including the expertise of the caregivers themselves, good accessibility and the professionals’ interest in the patient, their willingness to make appointments with each other and collaborate effectively and transparently, including through the use of joint documentation [9].

Our approach is also characterized by the fact that the care team itself, including the PRHC and their relatives, assumes responsibility for the components of the care concept. The need for *interprof* HOME can be reported to the nurse (*interprof* HOME agent) by all members of the interprofessional team, of which PRHC and relatives are integral members. However, the home care service pays particular attention to this special need as part of the already routinely conducted care consultation, which implies that existing resources (personnel structures and work processes) are used. It differs from many other German projects that are currently in progress where care and case managers (CCM) record the care needs of elderly patients [15–18]. For example, in the RubiN study [16, 17], patients receiving care from CCM in regional networks were very satisfied with their care, and as a result, the number of hospitalisations decreased. However, the measure has led to additional expenditures [16, 17]. In our view, the PRHC and their relatives should be empowered and supported with regard to their autonomy and their further needs by the *interprof* HOME agent and other healthcare providers. The current *interprof* HOME concept utilises specifically trained nurses as *interprof* HOME agents. In the long run, other professionals of the direct healthcare provider team could be involved. In Germany, this could be taken over by professionals with newly established roles such as physician assistants, advanced practice nurses, community health nurses or already established non-physician practice assistants. The *interprof* HOME care concept promotes a culture of person-centered care. PRHC can decide who coordinates their care in addition to the *interprof* HOME agent and are actively involved in communication (joint home meeting, dedicated telephone number, digital communication system) and joint care planning (before and during joint home meetings). Regular opportunities for sharing ensure access to care-related information (digital communication systmen) and address the needs of all involved. The term “team” refers to PRHC, their relatives, and various health professionals, fostering a common team identity for integrated collaboration. The concept encourages interprofessional collaboration with shared accountability and clearly defined roles and goals for all parties involved [64].

Altogether, in the view of the extensive empirical underpinning of the final *interprof* HOME concept, we are convinced, that the relevance of the developed components was confirmed. A validation of the concept and its components should take place by introducing it into healthcare practice, coupled with sound evaluation of acceptability, feasibility and clinical and resource-related outcomes. Plannings for such at trial are ongoing.

Finally, we assume that *interprof* HOME will improve the health-related quality of life and satisfaction of the PRHC with outpatient care and reduce unnecessary hospital admissions. More targeted remedies and home nursing care may even lead to lower costs. Following the guidelines of the MRC framework [11], we intend to pilot *interprof* HOME as a next step with regard to feasibility and acceptability and its further validation. In particular, we need to determine whether and how to ensure professionals’ participation in joint meetings and home visits. Additionally, consideration should be given to the remuneration of interprofessional cooperation and collaboration as well as to the formation of regional networks. In a subsequent randomised controlled trial, the impact of the refined *interprof* HOME should be analysed and evaluated, including analyses of processes and healthcare costs.

### Strengths and limitations

The main strength of the study is its participatory design. All the groups involved in home care have actively contributed to the development of the interprofessional person-centred care concept *interprof *HOME. By integrating their perspectives and needs, the acceptance of the concept in home healthcare is considered to be higher and its implementation in everyday life is more feasible [65, 66].

Moreover, the mixed methods approach allowed us to answer the research questions from different methodological angles and therefore provided deeper insight into the topic. The composition of the interprofessional research team is another strength. Constant discussions of the data and the analysis process in subgroups, along with regular meetings of the entire study team, led to comprehensive and careful consideration of the subject matter.

The fact that the sample only represents a group of PRHC and their relatives and professionals who were willing to participate (selection bias) and that persons with dementia, aphasia and other restricting conditions were not surveyed may be limiting factors. However, the perspective of relatives of PRHC with limited abilities to speak for themselves was taken into account.

## Conclusion

This study describes the rigorous development of an interprofessional person-centred care concept for PRHC using a transparent and systematic multistep process. It is expected that the intervention combining multiple components and targeting different interest holders - the PRHC, relatives and professionals - will improve care for PRHC provided by an interprofessional team more effectively. The components are aimed at more structured mutual exchange, more intensive communication, and more participation by the PRHC and their relatives in the planning and provision of care in line with their own goals and needs. The *interprof* HOME care concept is now ready for implementation and investigation of its feasibility and impact.

## Supplementary Information

Supplementary Material 1.

Supplementary Material 2.

## Data Availability

In addition to the survey data, we also collected data consisting primarily of transcripts of audio recordings of interviews, focus groups, observations and the expert workshop. We cannot share the original data because the consent forms state that the data will be viewed only by the study staff and used only for the purposes of the study.

## Abbreviations

CCM: Care and case managers
GP: General practitioner
OT: Occupational therapists
PT: Physiotherapists
PRHC: People receiving home care
SWOT: Strengths, Weaknesses, Opportunities, Threats
TIDieR: Template for intervention description and replication
WHO: World Health Organization
WP: Work package
