# Supplementary material for: Development of an interprofessional person-centred care concept for persons with care needs living in their own homes in Germany (interprof HOME): a mixed methods study
Source: BMC Prim Care. 2025 Nov 15;26:363. doi: 10.1186/s12875-025-03098-0 (PMC12619257; doi:10.1186/s12875-025-03098-0)
Supplement: Supplementary file 1 — Supplementary Material 1. [file 12875_2025_3098_MOESM1_ESM.docx]

**Additional file 1:** *Intervention protocol of interprof* HOME *according to TIDieR (template for intervention description and replication)* [1] - an interprofessional person-centred care concept for persons with care needs living in their own homes.

**1. Brief Name**

*interprof* HOME - an interprofessional person-centred care concept for persons with care needs living in their own homes.

**2. Why?**

Home care is often provided both by relatives and various professionals, so that constant mutual coordination is required. This rarely occurs in a systematic and structured manner. Inadequate collaboration and communication between the healthcare professionals involved can lead to "inconsistent care" [2] and have a negative impact on the safety of those in need of care, for example, due to medication errors. [3] In addition, patients have reported coordination problems associated with unplanned emergency room visits and hospitalizations. The overall aim of the mixed methods study was to develop a care concept to improve person-centred interprofessional collaboration for people receiving home care (PRHC) while systematically considering the perspectives of the PRHC, relatives, nurses of outpatient care services, general practitioners (GPs), occupational therapists, physiotherapists and speech therapists. The next step is to test and implement the *interprof* HOME care concept, that has been developed, in a feasibility study and a cluster-randomised controlled trial. Implementation of *interprof* HOME is expected to improve person-centred interprofessional collaboration for PRHC, so that their health-related care needs are met more effectively, thus leading to improvement of their health-related quality of life, participation and satisfaction with their home care. *interprof* HOME is also expected to decrease hospital admissions, unscheduled visits to primary care physicians, use of emergency medical and outpatient services and other healthcare services and reduce symptom burden, pain and the subjective burden of family caregivers.

**3./4. What (Materials/Procedures)?**

*interprof* HOME is considered a complex intervention, as it contains multiple components. A description of the characteristics (why, what, who, how, where, when and how much?) of the intervention components is described in Table S1. Several strategies are used to facilitate the implementation of *interprof* HOME. They are described in the following:

**Designation of an *interprof* HOME agent**

In each home care team^[[1]](#footnote-2)^ (PRHC, relative, nurse, GP, therapists if available), one nurse from an outpatient care service is designated as the *interprof* HOME agent and another one as a substitute. The tasks of these agents are to inform the respective care team (PRHC, relatives, GP, therapists) about the *interprof* HOME intervention and to initiate, coordinate and monitor the activities for the implementation of the components. The agents also stay in close contact with the care team. The nursing service management designates the interprof HOME agents. To be nominated as an *interprof* HOME agent or a substitute, nurses must fulfil each of the following criteria: 1. Have a qualification as a registered nurse (three-year vocational training in geriatric or general nursing), 2. Have worked for at least 20 hours per week on average, and have experience in conducting nurse consultations according to § 7a SGB XI Nursing Insurance Act for at least three months. They receive a training in advance about the circumstances, when *interprof* HOME is to be implemented and about their tasks.

**Handbook and documentation forms**

All participants receive a handbook containing descriptions of all the components and the documentation forms in order to prepare for the initial joint home visit.

**Online documents**

It is the *interprof* HOME agents' responsibility to send all the completed documentation forms to all members of the care team and place a printed document in the analogue *interprof* HOME folder in the PRHC’ s home.

**Paper-based *interprof* HOME folder in the PRHC’s home**

In order to make the relevant information accessible to all the persons involved, a paper-based folder is planned in addition to a digital one. Copies of all the completed documentation forms are stored there.

**5. Who provides?**

The *interprof* HOME agents and their substitutes are in charge of implementing and maintaining *interprof* HOME. Outpatient nursing service managers, PRHC and their relatives, GPs and therapists, are also involved in the implementation.

**6. How?**

The implementation may vary, depending on the component and the respective implementation strategy. Elements of the implementation strategies include regular contact by telephone or email and personal conversations between the study team and the *interprof* HOME agents, among others.

**7. Where?**

First, the PRHC is recruited via outpatient nursing services or general practices in Germany (Göttingen, Hamburg, Lübeck, Köln). All the nurses, GPs, relatives and therapists available for the participating PRHC are invited to take part in the study.

**8. When and how much?**

In order to implement all the intervention components of *interprof* HOME at least once, the implementation should be carried out for at least 6 months, preferably 12 months.

Table S1: Intervention components of *interprof* HOME - an interprofessional person-centred care concept for persons with care needs living in their own homes; PRHC = people receiving home care, GP = general practitioner

| **Intervention component** | **Designation and support of coordinator(s)** |
| --- | --- |
| **Why?** | Designation of coordinators should help the PRHC be more involved in assessing care needs and enhance their care through a holistic approach. Furthermore, the coordinator(s), supported by the other care providers, should help support the PRHC or reduce the burden on them and their relatives. |
| **What (materials)?** | To become an *interprof* HOME agent, those selected receive training in advance that qualifies them as a specialised *interprof* HOME agent, as well as a handbook containing all the measures and procedures and documentation forms. |
| **Who?** | The *interprof* HOME agent and a relative (possibly another carer) share the tasks. *Interprof* HOME agents normally are nurses who also provide care counselling to the person in need of care. |
| **How?** | The coordinators take over all the organizational tasks, such as organizing and picking up prescriptions, medications and remedies. Prior to the joint meetings, one of the coordinators conducts an interview with the PRHC to identify current health concerns and goals. The coordinators then plan the course of action for achieving these goals together with the care team. The other care team members support the coordinator as much as possible. |
| **Where?** | Conversations about concerns and goals between one of the coordinators and the PRHC take place by telephone, in online meetings or as face-to-face meetings at the PRHC’s home. Moreover, coordinators organize and take notes of the initial joint home visit and further meetings. |
| **When and how much?** | The coordinators are designated before the initial joint home visit. They are responsible for all the organizational tasks related to the PRHC’s care. About a week before the joint meetings, one of them ascertains the PRHC's concerns and goals. |

| **Intervention component** | **Initial joint home visit** |
| --- | --- |
| **Why?** | The aim of the initial joint home visit is to discuss the current healthcare needs of the PRHC and to jointly agree on procedures in the individual care team to improve the care situation. The initial joint home visit is also intended to strengthen collaboration within the care team by getting to know each other, actively planning how to proceed and starting or intensifying person-centred care and interprofessional collaboration. |
| **What (materials)?** | Prior to the initial joint home visit, the *interprof* HOME agent sends a handbook containing all the measures and procedures and documentation forms to all the members of the care team. |
| **Who?** | All the members of the care team may participate in the initial joint home visit. The PRHC decides if all the professional providers and/or relatives participate. |
| **How?** | If a PRHC requires specialised interprofessional home care, the need for *interprof* HOME can be declared to the nurse (*interprof* HOME agent) by everyone in the care team, but the home care service, in particular, pays attention to this specialised need as part of the care consultation. The *interprof* HOME agent organizes and facilitates the initial joint home visit. He or she arranges the appointment. The *interprof* HOME agent also reminds all the participants shortly before the initial joint home visit by messenger and/or by telephone. A preliminary discussion is held with the PRHC by the *interprof* HOME agent. The *interprof* HOME agent is requested to document the initial joint home visit by taking minutes using a documentation form. After the initial joint home visit, he or she sends the minutes to the care team by a communication system. A print version is placed in the *interprof* HOME folder at the PRHC’s home |
| **Where?** | The initial joint home visit is on-site. |
| **When and how much?** | The initial joint home visit takes place in the first month after the teams are admitted to the study and lasts no longer than 1 hour. |

| **Intervention component** | **Digital communication system (messenger)** |
| --- | --- |
| **Why?** | A digital communication system (messenger) makes it possible to exchange text messages, images and documents, thus enabling rapid transmission of symptoms and pain reports and the initiation of measures to prevent unnecessary use of medical services and hospital admissions. The messenger system also provides a site where all information can be bundled. Not only that, the initial joint home visit and the joint meetings can be scheduled by messenger. |
| **What (materials)?** | The *interprof* HOME agent documents the decisions regarding a messenger provider in a documentation form. |
| **Who?** | The *interprof* HOME agent coordinates the decision-making process with regard to the provider. |
| **How?** | All the members of the care team decide on a privacy-compliant messenger provider and the future users: all the members of the care team (variant A) or only all the professional providers (variant B). The *interprof* HOME agent sets up the messenger group account and invites other participants to join. If the PRHC cannot or does not want to use the messenger system, the coordinator manages communication by messenger on his or her behalf. |
| **Where?** | The members of the care team install the digital communication system (messenger) on their computer or (work) mobile phone. |
| **When and how much?** | Within the week after the initial joint home visit the *interprof* HOME agent set up a digital communication system. All the members of the care team use the appropriate documentation to provide their contact information (email address and/or phone number) to the *interprof* HOME agent, thus permitting him or her to invite them to the group. The *interprof* HOME agent provides the documentation form to the care team by messenger. A printed documentation form is stored in the *interprof* HOME folder in the PRHC’s home.  The *interprof* HOME agent uses the messenger system for non-urgent communication and to forward all the standard forms. The messenger system is also used to plan the joint home visit and joint meetings. It can also be used for joint video conference meetings if a corresponding license is available. |

| **Intervention component** | **Dedicated phone numbers** |
| --- | --- |
| **Why?** | The dedicated phone number makes it possible for all the members of the care team to contact each other more quickly. This enables them to respond faster in acute situations, thus preventing subsequent complications as well as unnecessary hospital admissions and use of emergency services. |
| **What (materials)?** | At the initial joint home visit, the *interprof* HOME agent uses a documentation form to record decisions on times and situations with regard to the dedicated phone number made by the care team. After the initial joint home visit, he or she makes the standard form available to the care team by messenger. A print version of the document is placed in the *interprof* HOME folder in the PRHC’s home. |
| **Who implements?** | The *interprof* HOME agent assists the individual care team with implementation. |
| **How?** | All the members of the care team set up a dedicated phone number, if not already available. The phone number, the times of availability and the situations where it will be used are discussed during the initial joint home visit. |
| **Where?** | All the professional providers set up a dedicated phone number within their facilities or a mobile phone number. |
| **When and how much?** | Within one week after the initial joint home visit, all the professional providers communicate their dedicated phone number, the times of availability and the situations where it will be used to the nurse using a documentation form. This number can be used to call the providers at the agreed times and in the agreed situations. |

| **Intervention component** | **Joint meetings** |
| --- | --- |
| **Why?** | By having all the participants agreement on goals and procedures during the initial joint home visit, the PRHC’s care can be tailored more precisely and thus improved. Joint evaluation of the achievement of goals at the next joint meeting is used to show changes to all the participants directly. |
| **What (materials)?** | The dates of the joint meetings along with the goals and agreed procedures for the meetings are recorded in a documentation form at the initial joint home visit. After the meeting, the standard form is sent to everyone by messenger and a copy is printed and placed in the *interprof* HOME folder. |
| **Who implements?** | All the members of the care team attend the joint meeting. |
| **How?** | The *interprof* HOME agent organizes and facilitates the joint meetings. The *interprof* HOME agent also reminds all the participants shortly before the joint meeting by messenger and/or by telephone. |
| **Where?** | The joint meetings take place online in a video conference or by telephone. |
| **When and how much?** | Joint meetings are held on an as-needed basis. |

**References**

1. Hoffmann TC, Glasziou PP, Boutron I, Milne R, Perera R, Moher D, et al. Better reporting of interventions: template for intervention description and replication (TIDieR) checklist and guide. BMJ. 2014;348:g1687.
2. Görres S, Warfelmann C, Meinecke P, et al. Perspektivenwerkstatt Patientensicherheit in der ambulanten Pflege. Abschlussbericht für das Zentrum für Qualität in der Pflege (ZQP) 2018.
3. Berland A, Bentsen SB. Medication errors in home care: a qualitative focus group study. *1365-2702* 2017;26(21-22):3734–41.

1. When we use the term "team", we are aware of the fact that the PRHC, relatives and various healthcare professionals may not yet have formed a team that shares a team identity and collaborates in an integrated and interdependent manner to solve problems and provide services at the beginning of the implementation phase. The interprofessional care concept should reinforce interprofessional collaboration so that there is a sense of shared accountability and interdependence among the participating individuals and the roles/goals are clear. The PRHC should be integral members of the team. [↑](#footnote-ref-2)
