# Supplementary material for: Development of an interprofessional person-centred care concept for persons with care needs living in their own homes in Germany (interprof HOME): a mixed methods study
Source: BMC Prim Care. 2025 Nov 15;26:363. doi: 10.1186/s12875-025-03098-0 (PMC12619257; doi:10.1186/s12875-025-03098-0)
Supplement: Supplementary file 2 — Supplementary Material 2. [file 12875_2025_3098_MOESM2_ESM.pptx]

## Slide 1
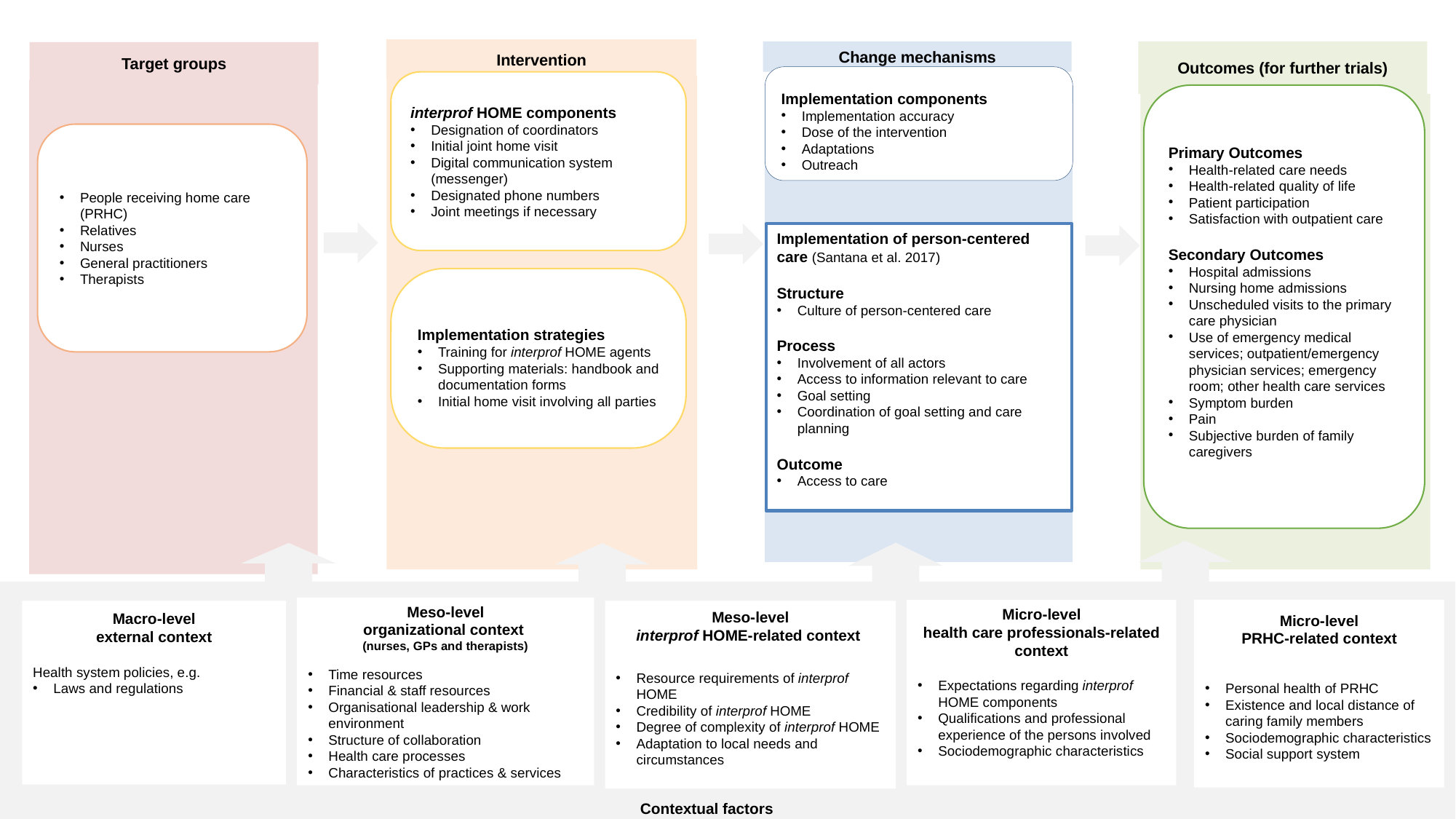

Intervention
Outcomes (for further trials)
Change mechanisms
Target groups
Implementation components
Implementation accuracy
Dose of the intervention
Adaptations
Outreach
interprof HOME components
Designation of coordinators
Initial joint home visit
Digital communication system (messenger)
Designated phone numbers
Joint meetings if necessary
Primary Outcomes
Health-related care needs
Health-related quality of life
Patient participation
Satisfaction with outpatient care
Secondary Outcomes
Hospital admissions
Nursing home admissions
Unscheduled visits to the primary care physician
Use of emergency medical services; outpatient/emergency physician services; emergency room; other health care services
Symptom burden
Pain
Subjective burden of family caregivers
People receiving home care (PRHC)
Relatives
Nurses
General practitioners
Therapists
Implementation of person-centered care (Santana et al. 2017)
Structure
Culture of person-centered care
Process
Involvement of all actors
Access to information relevant to care
Goal setting
Coordination of goal setting and care planning
Outcome
Access to care
Implementation strategies
Training for interprof HOME agents
Supporting materials: handbook and documentation forms
Initial home visit involving all parties
Meso-level
organizational context
(nurses, GPs and therapists)
Time resources
Financial & staff resources
Organisational leadership & work environment
Structure of collaboration
Health care processes
Characteristics of practices & services
Micro-level
PRHC-related context
Personal health of PRHC
Existence and local distance of caring family members
Sociodemographic characteristics
Social support system
Micro-level
health care professionals-related context
Expectations regarding interprof HOME components
Qualifications and professional experience of the persons involved
Sociodemographic characteristics
Macro-level
external context
Health system policies, e.g.
Laws and regulations
Meso-level
interprof HOME-related context
Resource requirements of interprof HOME
Credibility of interprof HOME
Degree of complexity of interprof HOME
Adaptation to local needs and circumstances
interprof ACT - Quantitative Prozessevaluation
Contextual factors
